# Mast Cells as a Target—A Comprehensive Review of Recent Therapeutic Approaches

**DOI:** 10.3390/cells12081187

**Published:** 2023-04-19

**Authors:** Joanna Baran, Anna Sobiepanek, Anna Mazurkiewicz-Pisarek, Marta Rogalska, Aleksander Gryciuk, Lukasz Kuryk, Soman N. Abraham, Monika Staniszewska

**Affiliations:** 1 Centre for Advanced Materials and Technologies CEZAMAT, Warsaw University of Technology, 02-822 Warsaw, Poland; joanna.baran@pw.edu.pl (J.B.); marta.rogalska@pw.edu.pl (M.R.); aleksander.gryciuk@pw.edu.pl (A.G.); 2 Faculty of Chemistry, Warsaw University of Technology, 00-664 Warsaw, Poland; anna.sobiepanek@pw.edu.pl; 3 Department of Virology, National Institute of Public Health NIH-NRI, 00-791 Warsaw, Poland; 4 Department of Pathology, Duke University Medical Center, Durham, NC 27710, USA; soman.abraham@duke.edu

**Keywords:** mast cells, cytokines, degranulation, anaphylaxis, cancer, therapy

## Abstract

Mast cells (MCs) are the immune cells distributed throughout nearly all tissues, mainly in the skin, near blood vessels and lymph vessels, nerves, lungs, and the intestines. Although MCs are essential to the healthy immune response, their overactivity and pathological states can lead to numerous health hazards. The side effect of mast cell activity is usually caused by degranulation. It can be triggered by immunological factors, such as immunoglobulins, lymphocytes, or antigen–antibody complexes, and non-immune factors, such as radiation and pathogens. An intensive reaction of mast cells can even lead to anaphylaxis, one of the most life-threatening allergic reactions. What is more, mast cells play a role in the tumor microenvironment by modulating various events of tumor biology, such as cell proliferation and survival, angiogenesis, invasiveness, and metastasis. The mechanisms of the mast cell actions are still poorly understood, making it difficult to develop therapies for their pathological condition. This review focuses on the possible therapies targeting mast cell degranulation, anaphylaxis, and MC-derived tumors.

## 1. Introduction

Mast cells (MCs) are evolutionarily old cells that genesis dates back to the first immune mechanisms in organisms of the urochordate genus. Despite their relatively old discovery and description by Paul Ehrlich in 1876, these cells’ mechanisms of immunological interactions are still not fully understood [1]. MCs are derived from multipotential stem cells in the bone marrow and yolk sac [2]. Immature cells (CD34^+^, c-kit^+^, Ly-1^+^, CD14^−^, and CD17^−^) circulate throughout the body and migrate into tissues, often adjacent to blood vessels and close to epithelial surfaces, creating a barrier for pathogens (e.g., in the gastrointestinal tract, skin, and respiratory epithelium) [3]. Under normal conditions, mature mast cells reside in the peripheral tissues, differentiating into functional forms. The phenotype of mast cells differs depending on the microenvironments they are in, adjusted to the functions they serve, such as participating in innate and adaptive immune responses to pathogens [4]. In injured or infected tissues, MCs regulate inflammation, working both ways—by amplifying or suppressing the process [5]. Despite their important role in healthy immune responses, MCs have been implicated in multiple diseases, such as mastocytosis, mast cell activation syndrome (MCAS), osteoporosis, autoantibody-mediated arthritis, multiple sclerosis, allergic reactions, and numerous lung pathophysiologies. As their role as a therapeutic target remains elusive, this review focuses on the possible therapies connected to dysfunctioning mast cells and current drugs presenting activity towards them.

## 2. Mast Cells’ Receptors and Mediators

**Figure 1:**
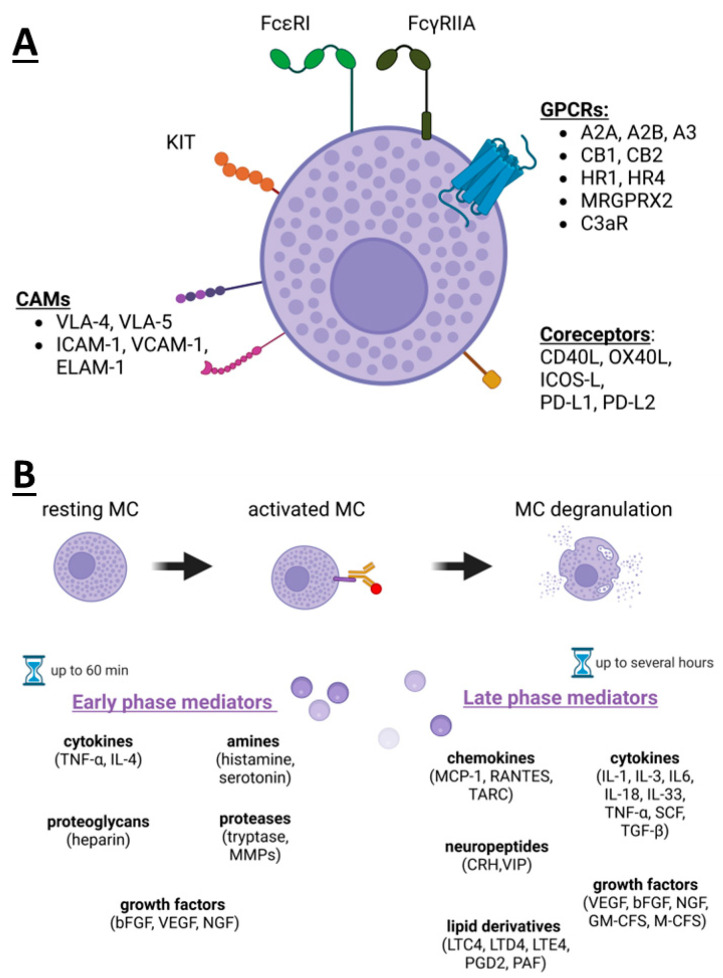
Several types of receptors and coreceptors are present on the mast cell surface (**A**) and upon MCs activation, various mediators of the early and late phases are released (**B**). Figure created with BioRender.com.

Mast cells are the immune cells distributed throughout nearly all tissues, mostly in the skin, near blood vessels and lymph vessels, nerves, and in the lungs and the intestines. These cells express numerous groups of surface receptors (with high/low affinity for the allergen immunoglobulin E—FcεRI and FcγRIIA receptors; KIT receptor with affinity to the stem cell factor; as well as G protein-coupled receptors (GPCRs): adenosine receptors—A2A, A2B, and A3, cannabinoid receptor type 1 and 2 (CB1 and CB2), histamine receptors type 1 and 4 (H1R, H4R), mass-bound X2 G protein-coupled receptor (MRGPRX2), and complement component C3a receptor (C3aR)), by which they can be stimulated to certain actions (release of specific intracellular mediators, but also to the total mast cell degranulation) (Figure 1A). On the other hand, several cell adhesive receptors (CAMs) and coreceptors enable their binding to different cells, tissues, and surfaces. Mast cells store a wide spectrum of biologically active mediators that may have a potential positive or negative effect on various target cells. Upon activation, mast cells within minutes can release mediators, which were accumulated inside the cell; however, mast cells may likewise be stimulated to produce de novo mediators and release them from the cells even several hours after activation (Figure 1B). Some of these mediators are cytokines (e.g., Interleukin (IL)-1, IL-3, IL-6, IL-18, IL-33, Tumor Necrosis Factor (TNF)-α, Stem Cell Factor (SCF), Transforming Growth Factor (TGF)-β), chemokines (like Monocyte Chemoattractant Protein (MCP)-1, Regulated on Activation, Normal T-cell Expressed and Secreted (RANTES), Thymus and Activation-Regulated Chemokine (TARC)), growth factors (i.e., Vascular Endothelial Growth Factor (VEGF), basic Fibroblast Growth Factor (bFGF), Nerve Growth Factor (NGF), Granulocyte-Macrophage Colony-Stimulating Factor (GM-CSF), Macrophage Colony-Stimulating Factor (M-CSF), proteases (tryptase, Matrix Metalloproteinases (MMPs)), proteoglycans (heparin), amines (histamine, serotonin), neuropeptides (Corticotropin-Releasing Hormone (CRH), Vasoactive Intestinal Peptide (VIP)), and lipid derivatives (including Leukotriene (LT) C4, D4, and E4 (LTC4, LTD4, LTE4, respectively), Prostaglandin D2 (PGD2), Platelet-Activating Factor (PAF)) [6,7].

Mast cell degranulation can be monitored via various assays, including ELISA tests, flow cytometry, or colorimetric assays. ELISA tests are popular in the case of cytokines, chemokines, growth factors, and proteins, but as they use antibodies, the procedure is rather long. With flow cytometry, three approaches to detect mast cell degranulation predominate: detecting the appearance or upregulation of surface biomarkers (e.g., CD63 and CD107a), measuring changes in the mediator content (like histamine, chemokine (C-C motif) ligand 4, (CCL4), and chemokine (C-X-C motif) ligand 8 (CXCL8)), and staining of intracellular calcium ions. To enable the detection of degranulating cells in this method, fluorescently labeled antibodies or other labeled compounds with fluorochromes must be applied [8]. The colorimetric assays are easier, quicker, and can be applied for the most common mediators monitored during mast cell degranulation such as histamine, β-hexosaminidase (β-hex), or tryptase. However, the half-life of histamine released from cells is very short, thus its determination may not be as accurate [9] as the measurement of β-hexosaminidase and tryptase. To gain the information upon occurring mast cell degranulation, the release of a selected mediator to the supernatant is compared to the concentration of the mediator in the cell lysate. The main difference between these methods is associated with the amount of received information about the degranulating cells. The flow cytometry can show individual cells undergoing degranulation, whereas ELISA tests, as well as the colorimetric assays, focus on the average cells.

Several types of models can be applied for the investigation of mast cell degranulation. The in vitro studies include primary human and animal mast cells from the skin, the bone marrow-derived mast cells (BMMCs) or the peripheral blood mononuclear cells (PBMCs), as well as human and animal commercial cell lines [10]. During the in vivo studies, the most popular organisms are mice, rats, and pigs. However, several studies presented results on different models and may significantly differ. The same compound/drug may inhibit mast cell degranulation in one model but not in another model [11]. Thus, it is important to check the influence of potential drugs on various models.

Mast cells are involved in many physiological and pathological processes, such as wound healing, inflammation, angiogenesis, and tumor progression. For example, they have an impact on every stage of the wound healing process, from triggering an inflammatory reaction (histamine release), restoring the epidermis (proliferation and migration of keratinocytes) and blood vessels, to distributing collagen in the damaged tissues (stimulation of fibroblasts to produce extracellular matrix) [12]. Although there is no doubt that mast cells are also a crucial element of the tumor progression process, the described data provide conflicting results on whether mast cells play a pro- or anti-tumorigenic role. One can distinguish mediators with properties that stimulate (e.g., tryptase, IL-6/-8/-13, PAF, TGF-β, VEGF-α/-β, FGF-2) and inhibit (like histamine, heparin, IL-4/-9, TNF-α) cancer development [13]. For now, this question remains unanswered [6].

## 3. Mast Cells Degranulation and Its Inhibitors

**Figure 2:**
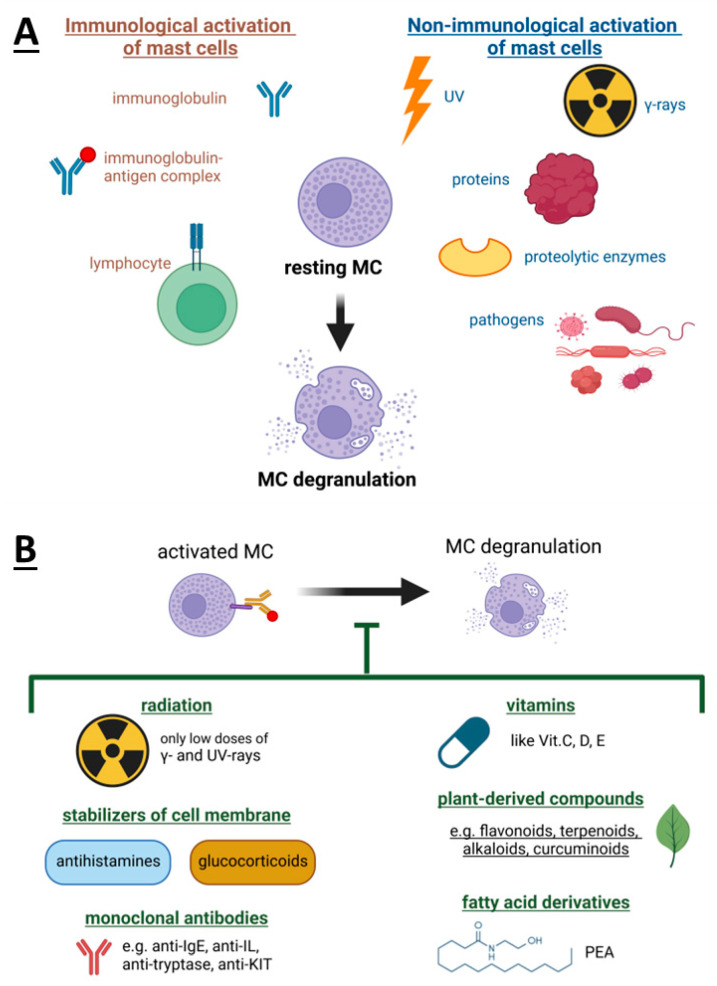
Mast cell degranulation can be stimulated by non-immunological and immunological factors (**A**), but this process can be also inhibited by various groups of compounds and factors (**B**). Figure created with BioRender.com.

Mast cell degranulation may occur due to one of the two mechanisms: non-immunological activation of cells (caused by radiation, pathogens, proteins, proteolytic enzymes, opioids, estrogens, and androgens) or immunological activation (generated by immunoglobulins, lymphocytes, and immunoglobulin–antigen complexes) (Figure 2A). Apart from symptoms like itching, swelling, or redness of the tissue, mast cell degranulation may also lead to anaphylaxis, where the three most common causes are triggered by foods (milk, peanuts, wheat, and soy), venom (bees, wasp, snakes), and medications (antibiotics, vaccines, painkillers, opioids, chemotherapy) [14]. In the following sections, several inhibitors of the mast cell degranulation process will be described (Figure 2B).

Several environmental factors may influence mast cell degranulation. Low doses of γ-ionizing radiation (<0.1 Gy) may function as an inhibitor of mast cell activation by suppressing the expression of FCεRI receptor and thus by suppressing the release of several mediators like histamine, β-hex, IL-4, and TNF-α. However, higher doses of this radiation (>0.5 Gy) induced apoptosis in RBL-2H3 rat cells [15]. Tissue exposure to ultraviolet (UV) radiation can also suppress cell-mediated immunity. For example, the number of MCs in the human skin areas exposed to sunlight (mainly from A and B bands) was much higher than in the sun-protected areas. The in vitro and ex vivo studies presented the increased release of mediators from mast cells. Although, UVA up to 25 J/cm^2^ and UVB 100 mJ/cm^2^ had no negative influence on the non-activated mast cells, for the activated mast cells (with IgE, SP, 48/80) significant reduction in histamine release was observed [16]. Thus, radiation may be used for phototherapy, in which the effect on the inflammatory disease is mediated by suppressing mast cells [15]. However, one must bear in mind that each exposure to UV radiation increases the risk of skin cancers’ appearance. In particular, UV radiation is one of the main causes of basal and squamous cell carcinoma, as well as melanoma. Their metastasis occurrence significantly reduces the chances of patients’ survival, even if the primary cancerous tissue is removed or if appropriate therapy is applied (melanoma cells frequently gain drug resistance) [17,18].

Mast cell stabilizers prevent cell degranulation by the stabilization of the cell membrane, and thus the release of multiple mediators is blocked. Such drugs are sodium cromoglycate (internasal format), nedocromil (for inhalation and topical ophthalmic, FDA-approved in the USA), ketotifen, pemirolast, and olopatadine (topical antihistamine). The last drug belongs to a well-known group of compounds intending to minimize histamine release to the organism [19]. Taking into consideration that almost all cells react directly to histamine appearance by the ligand–receptor interaction (especially in the highly inflamed areas) [12] and its occurrence influences the biological functions of these cells (e.g., increased proliferation of keratinocytes in the wound healing process, [20]; and increased migration of eosinophils by the extensive occurrence of adhesion molecules on the surfaces of these cells [21]), the disorder in the histamine receptor binding may help to achieve homeostasis of the immune system. The Older- and Newer-generation of Antihistamines (OgenAs and NgenAs, respectively) can be used to competitively occupy the histamine 1 receptor site in the cells, and by that, preventing the ligand–receptor binding [19]. The chemical structure of antihistamines is similar to histamine, and thus these compounds can be grouped into derivatives of ethanolamine, ethylenediamine, phenothiazine, piperazine, or propylamine. On the other hand, glucocorticoids also suppress the inflammatory functions of mast cells by stabilizing cell membranes [22]. Although the reduced number of mast cells was observed in airway mucosal biopsy specimens from humans with mild atopic asthma due to budesonide (glucocorticosteroid) application [23], these drugs generally presented a minimal effect on inhibiting mast cell degranulation. Incubation of mast cells with steroids did not alter the release of histamine, prostaglandin D2, or leukotriene C4; however, they were able to inhibit the production of some proinflammatory cytokines like IL-1, IL-6, and TNF-α [22].

The developed monoclonal antibodies target multiple aspects of mast cell functions and thus may be considered promising drugs, even though most of them are still in clinical trials. The first available monoclonal antibodies were used to reduce the levels of free IgE in serum, which can further downregulate the presents of the FcεRI receptor on mast cells and basophils. Thus, these drugs act by eliminating the activation signal for mast cells to begin the degranulation. Omalizumab is an already FDA-approved drug targeting IgE, whereas ligelizumab is still in clinical trials. An antibody with a different mechanism of action is Dupilumab, an FDA-approved drug, which is best known for its suppressive properties towards the IL-4 and IL-13 signaling. A novel antibody tested in clinical trials is MTPS9579A. It is directed against active tryptase, which is released from mast cells in high numbers during the degranulation process. In this case, the activity of released tryptase is limited by the dissociation of its tetrameric form into inactive monomers. A different approach was taken with the antibody CDX-0159 (in clinical trials) that is acting as an allosteric inhibitor of the c-kit/CD117 receptor present on the surface of mast cells. In general, it reduces the SCF binding and KIT tyrosine phosphorylation [24].

Some vitamins are listed as the first-line drugs for controlling mast cell degranulation. Vitamin C plays a significant role in the increased degradation of histamine, because its action is mainly based on the inhibition of histidine decarboxylase activity, which decreases the formation of histamine in the body [25]. The benefits of vitamin D supplementation are also well visible in several health conditions, including immunological diseases. Treatment with vitamin D can regulate the release of several specific cytokines and chemokines exerting anti-inflammatory action. For example, it promotes the augmentation of IL-4 and IL-10, and also inhibits the generation of IFN-γ and TNF-α [26]. This is because vitamin D binds to the gene transcription apparatus to regulate the gene expression of selected mediators, like TNF-α [27]. Moreover, mast cell proliferation, secretion, and survival can be inhibited by vitamin E supplementation [28], and vitamin B reduces phospholipase A-induced mast cell degranulation by limiting histamine release from cells [29]. Nevertheless, supplementation with vitamins should be strictly controlled as their too-high levels may have a negative influence on the body.

Various plants are rich in bioactive constituents which may also control mast cell degranulation. Some of the investigated plant-derived groups are flavonoids, stilbenes, phenanthrenes, diarylheptanoids, 3-phenyl-isocoumarins, phenylpropanoids, beta-carboline-type alkaloids, sesquiterpenes, and meroterpenes [30]. Flavonoids are polyphenolic compounds presenting various biological effects on cells, like cytoprotective, antioxidant, and anti-inflammatory properties. For example, fisetin, kaempferol, myricetin, quercetin, and rutin inhibited histamine release from the IgE- or PMA- and calcium ionophore A23187-activated RBL-2H3 rat mast cells. The intracellular calcium ions were also significantly inhibited by the cell treatment with these flavonoids. A decrease in the gene expression and production of the proinflammatory cytokines (TNF-α, IL-1β, IL-6, IL-8) was observed in the activated and flavonoid-treated mast cells. Thus, the potential treatment of allergic inflammatory disease can be obtained through the downregulation of mast cell activation by flavonoid treatment [31]. Furthermore, luteolin, fisetin, and diosmetin presented a significant reduction of β-hex release in the antigen-stimulated RBL-2H3 cells, whereas apigenin, luteolin kaempferol, and quercetin presented inhibition of TNF-α and IL-4 release [30]. Resveratrol, occurring in the skin of red grapes, exerts several beneficial properties, including anticancer and anti-inflammatory effects. It inhibited the IgE-mediated release of mast cells mediators like histamine, β-hex, leukotrienes, and prostaglandin D in a dose-dependent manner, and it suppressed passive cutaneous anaphylaxis reaction in IgE-sensitized mice [32]. Salvinorin A, a highly selective kappa agonist, is a diterpenoid present in the leaves of psychedelic sage. It can inhibit mast cell degranulation observed as the reduction in the release of β-hex, histamine, IL-4, and TNF-α [33]. Rhizoma coptidis is a herbal medicine containing several alkaloids, like berberine, coptisine, palmatine, and jatrorrhizine. Coptisine exhibits strong anti-inflammatory properties as it markedly decreased the levels of β-hex, histamine, IL-4, and TNF-α in RBL-2H3 rat cells. Furthermore, it inhibited granule release from mast cells and the F-actin cytoskeleton reorganization in these cells [34]. Cryptotanshinone extract from the medicinal herb *Salvia miltiorrhiza Bunge* belongs to the tanshinone group of bioactive compounds. It effectively mitigated the secretion of pro-inflammatory cytokines, including TNF-α and IL-1β, in IgE-activated mast cells. The effect was mediated by the inhibition of tyrosine kinase-dependent degranulation signaling pathways involving spleen tyrosine kinase and Lyn [35]. From the several known curcuminoids, only curcumin, monomethylcurcumin, and bisdemethoxycurcumin exhibited potential inhibitory activities towards mast cell degranulation observed as a reduction of β-hex release [30]. Curcumin, the main active ingredient of curry spice turmeric, has high anti-inflammatory properties. Its protective effect is most probably mediated by the suppression of the activated mast cells (inhibition of IL-4 and TNF-α production). The activated murine bone marrow-derived mast cells also exhibited elevated levels of β-hex compared with inactivated controls. Thus, curcumin may have the capacity to regulate the allergic responses in the organism [36].

So far, a few derivatives of fatty acids, in particular ethanolamines, were also described as inhibitors of mast cell degranulation. These compounds typically are present in human and animal organisms and regulate various cell/organism functions. Docosahexaenoyl ethanolamide (DHEA) is a metabolite produced in humans from docosahexaenoic acid (DHA), a long-chain polyunsaturated fatty acid. DHEA mitigates IgE-mediated degranulation of mast cells by decreasing the release of β-hex and calcium ions influx from RBL-2H3 rat cells. It also suppresses the IgE-mediated passive cutaneous anaphylaxis reaction in mice [37]. Anandamide (AEA), one of the most well-studied endocannabinoids, inhibits FcεRI-dependent mast cell degranulation and cytokine synthesis through the activation of two receptors (the cannabinoid receptor 2, CB2; and G protein-coupled receptor 55, GPR55). Moreover, AEA prevented the intracellular calcium ions increase in BMMC cells treated with IgE and dinitrophenol coupled to human serum albumin [38]. However, AEA did not reduce the activation of rat mast cells RBL-2H3 [39]. In terms of ethanolamine used the most frequently and with the best results for regulating mast cell degranulation, palmitoylethanolamide (PEA), an endocannabinoid-like compound, should be pointed out [11]. PEA reduced mast cell activation associated with the inflammation process by decreasing the antigen-evoked serotonin release from RBL-2H3 cells [39]. It was also able to counteract RBL-2H3 rat mast cell activation by substance P due to limiting histamine and β-hex release [40], as well as inhibiting the release of nerve growth factor (NGF) by the ester phorbol (PMA)-activated mast cells. As NGF is a member of the neurotrophin family and plays an important role in the stimulation of the angiogenesis process, PEA is believed to possess pro-angiogenic properties and may be considered for treatment in disorders characterized by prominent inflammation [41].

Most of the described compounds (drugs) limiting mast cell degranulation are summarized in Table 1.

## 4. Mast Cells as a Therapeutic Target in Allergic Inflammation

Mast cells exhibit a great potential to produce mediators playing a key role in allergic-related inflammatory diseases. Among them, we differentiate biogenic amines, proteoglycans, lysosomal enzymes, lipid mediators, cytokines, growth factors, mitogens, chemokines, etc. [42]. Allergic diseases, such as asthma, allergic rhinitis, atopic dermatitis, and others, despite occurring in different organs, share similar mechanisms.

Anaphylaxis is one of the most life-threatening and intensive allergic reactions. Unlike anaphylactoid reaction, it is an immunoglobulin E-mediated response [43]. Its symptoms can occur in multiple organ systems, such as cutaneous, respiratory, cardiovascular, and others. Mast cells together with basophils are the first cells that are responding to IgE-mediated anaphylaxis. Exposure to the factors, such as food, drugs, or plants, causes a sequence of events resulting in severe symptoms. However, according to clinical studies, in most cases the cause of anaphylaxis is undetermined (idiopathic anaphylaxis) [44]. The general mechanism of this process starts with allergen-specific IgE antibodies binding to high-affinity Fc receptors (FcεR1) present on the MCs surface. This leads to activation and degranulation in connective or mucosal tissues in which they reside. During degranulation, MCs release mediators such as histamine, leukotrienes, prostaglandins, cytokines, proteases, kinases, and nitric oxide. After some time, other cells of the immune system join the response stimulated by mediators. In this group, special attention should be paid to eosinophils participating in allergic inflammation [45]. Their reaction is induced by allergen-specific T helper 2 cells and Th2 cytokines [46]. Eosinophil response to inflammation state can lead to eosinophilia, intensifying the inflammation even more, and thus increasing the intensity of the symptoms of anaphylaxis.

**Figure 3:**
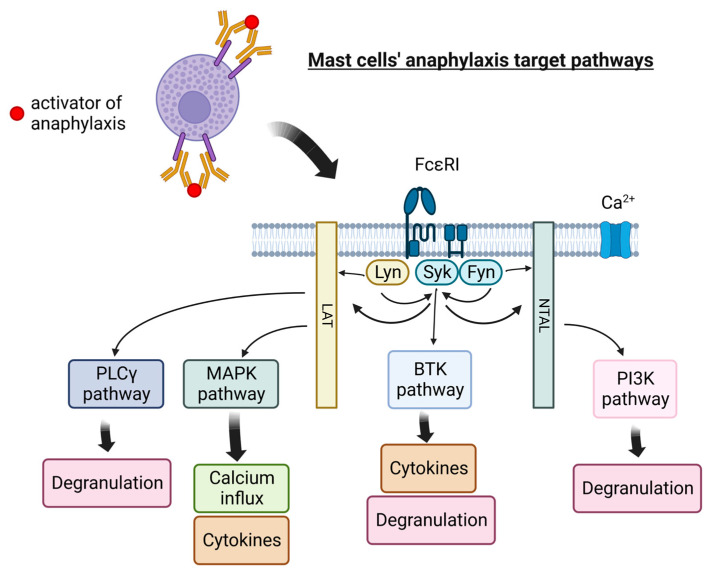
Mast cells’ pathways targeted in anaphylaxis therapy. PLCγ—Phosphoinositide-specific phospholipase C, MAPK—mitogen-activated protein kinases, BTK—Bruton’s tyrosine kinase, LAT—linker for activation of T cell, NTAL—non-T cell activation linker, PI3K—phosphoinositide 3-kinase, Lyn—Lyn kinase (Src family), Syk—Syk tyrosine kinase, Fyn—Fyn tyrosine kinase, FcεRI—a high-affinity IgE receptor. Figure created with BioRender.com.

Currently, therapeutic options for the treatment of idiopathic anaphylaxis are limited with variable efficacy. Popular targets for anaphylaxis management are shown in Figure 3.

Epinephrine is the first-choice medicine for anaphylaxis [47]. It prevents hypotension and laryngeal edema, the life-threatening symptoms of the reaction. Epinephrine is one of the adrenergic drugs acting on several receptors. Its action on the α1 receptor increases vasoconstriction and peripheral vascular resistance while reducing mucosal edema. The effect on the α2 receptor reduces the release of insulin and norepinephrine. Epinephrine also acts on β1 receptors to increase inotropy and chronotropy, and β2 receptors to reduce mediator release and increase bronchodilation, vasolidation, and glyconeolysis [48]. Although epinephrine has many advantages, some patients are showing symptoms refractory to epinephrine, such as bronchospasm. In those cases, 2-agonists, e.g., albuterol, can be used [49]. Patients with respiratory symptoms can also benefit from high-flow oxygen. The epinephrine injection is often followed by further symptom management. At this point, corticosteroids and antihistamines apply.

Monoclonal antibodies are a novel approach in the treatment of anaphylaxis symptoms. In the group consisting of omalizumab, benralizumab, reslizumab, mepolizumab, and dupilumab, only dupilumab showed a negative signal for anaphylaxis [50]. Dupilumab is a humanized IgG monoclonal antibody that binds to IL-4Rα, blocking IL-4 and IL-13 intracellular signaling. In the newest case report, dupilumab was effective in preventing recurrent anaphylaxis and in treating severe asthma [51].

Inhibiting FcεRI-mediated signaling is one of the approaches that could be efficient in preventing anaphylaxis. The studies show that Sirtuin 6 can act as a negative regulator in this signaling pathway; therefore, it can be considered as a new therapeutic strategy for anaphylaxis [52]. There exists a considerable body of literature on Bruton’s tyrosine kinase (BTK), which is an enzyme essential for high-affinity IgE receptor (FcεRI) signaling. To address BTK inhibitors’ efficacy against anaphylaxis, several FDA-approved substances were tested. Ibrutinib (Ibrivuca) is a drug that is FDA-approved for the treatment of mantle cell lymphoma, chronic lymphotic leukemia, and Waldenstroms macroglobulinemia. In the clinical studies completed in 2018 (NCT03149315), it was tested in 2 standard doses to check if it can limit allergic reactions. The studies showed reduction/elimination of skin-prick-test activity to allergens in healthy allergic adults. Acalabrutinib (Calquence) (NCT05038904) and Tirabrutinib (Velexbru) (NCT04947319) are other drugs from the group of BTK inhibitors currently tested to prevent anaphylaxis, approved or in the process of FDA approval for tumor treatment. The method introduced by Dispenza et al. has the advantage that they used an injection of human CD34+ cells into NSG-SGM3 mice, thus obtaining a model for testing the inhibition of anaphylaxis by BTK inhibitor drugs [53]. Studies on mice confirm the effectiveness of these drugs in a new application and are the basis for further research. In the research from 2022 [54], the authors suggested that BTK inhibitors decrease mast cell degranulation and hypothermia, but not in all of the conditions applied. In the same paper, they implied that a combination of histamine receptor 1 antagonists, β-adrenergic agonists, and a spleen tyrosine kinase (Syk) inhibitor could completely inhibit IgE-mediated hypothermia.

Several natural active compounds show great potential in managing anaphylaxis symptoms. Anemoside B4 is a triterpenoid abundant in the roots of *Pulsatilla chinensis* [55]. In both in vitro and in vivo studies, it was noted that Anemoside B4 can limit (IgE)-mediated allergic responses by inhibiting cell degranulation, calcium influx, and PLC/IP3 and JAK/STAT3 phosphorylation. Fucoxanthin is another natural compound with promising anti-anaphylactic properties [56,57]. The studies show that the use of fucoxanthin allows the management of cytokine production, the induction of cell survival molecule NF-κB p65, and the phosphorylation of IκBα. It was also proven that fucoxanthin could repress the allergic rhinitis induced by OVA (albumin from chicken egg white). Benzoylpaeoniflorin is a cage-like monoterpenoid glycoside isolated from *Paeonia lactiflora* extract [58]. It shows anti-anaphylactic activity through the mechanism based on the inhibition of HDC (Histidine decarboxylase, crucial in histamine synthesis pathway) and MAPK signal pathways. Ginsenosides are active steroid compounds, first isolated in 1963 from ginseng. The studies have emphasized that ginsenoside Rh2 (G-Rh2) reduced calcium uptake and histamine release [59]. The suggested mechanism included blocking IgE-induced degranulation by inhibiting AKT-NrF2 and p38MAPK-Nrf pathways. Studies of G-Rg3 have shown that it can decrease histamine release from MCs by enhancing cAMP levels and calcium influx, which is the opposite effect to Rh2. Protection against anaphylaxis is seen here in the regulation of mitogen-activated and receptor-interacting kinases in mast cells [60]. Previous research showed that G-Rh2′s antiallergic activity can be exhibited by stabilization of cell membrane and inhibiting of NO and PGE2 production [58,61]. Some authors have also suggested that ginsenoside Rh1 possesses antiallergic activities. However, in this case, its effectiveness was sought in the inhibition of mast cell degranulation thanks to cell membrane-stabilizing and anti-inflammatory activities [62]. Although studies have been conducted by many authors, the mechanism of ginsenosides is still insufficiently explored.

Another approach to managing anaphylaxis is focusing on Lyn kinase inhibition. Lyn kinase regulates phosphorylation of the protein scaffolds LAT (linker for activation of T cell) and NTAL (Non–T cell activation linker). Lyn is the first protein kinase phosphorylated and activated after allergen-IgE antibody complexes crosslink with FcεRI on the surface of MCs [63,64]. Alpha-linolenic acid is a substance that can modulate allergic reaction and MCs degranulation by inhibiting Lyn kinase activity, which was proven in vitro using LAD2 cells, and in vivo using OVA (albumin from chicken egg white)-stimulated mice model [65]. A similar mechanism for preventing anaphylaxis is described in Ashikari’s article from 2022 [66]. This time, salicylaldehyde was tested in vivo on the passive anaphylaxis mouse model. Studies have shown that the use of this substance reduced the symptoms of anaphylaxis in mice, such as increased temperature in the case of systemic anaphylaxis and swelling and vascular permeability in cutaneous anaphylaxis.

It is evident that the complex interactions between immune cells and structural cells in the inflammatory microenvironment determine how allergic reactions shift out. A similar situation is present in the microenvironment of cancer, where the immune system plays a crucial role [46].

A summary of the drugs for anaphylactic shock is presented in Table 2.

## 5. Mast Cell-Targeted Strategies in Cancer Therapy

For many years, mast cells were mainly known for their role in allergic reactions; however, the last decade has brought many new studies on these cells. The results clearly indicate that these cells also participate in the development of cancers, including hematopoietic cancers [68,69]. Mast cells play a multifaceted role in the tumor microenvironment by modulating various events of tumor biology, such as cell proliferation and survival, angiogenesis, invasiveness, and metastasis. Moreover, tumor-associated mast cells have the potential to shape the tumor microenvironment by establishing crosstalk with other tumor-infiltrating cells. MCs could stimulate the growth, neo-angiogenesis, and metastasis of tumors by multiple mechanisms [70,71,72,73].

Mast cells can exhibit anti-tumor activity either through: (a) direct tumor cell cytotoxicity and release of tumor necrosis factor or indirectly via mast cell-released heparin actions on fibroblasts, (b) acting as sentinel cells that secrete multiple chemokines that mobilize anti-tumor immune effector cells to tumor sites, (c) modulating immune effector cell responses and differentiation through the release of cytokines or through cell–cell interactions [12,72,74].

On the other hand, MCs also exhibit pro-tumor effects. Activated mast cells can potentiate the deregulated tissue of the tumor microenvironment and favor tumor growth, and spread through: (a) the release of pro-angiogenic factors which enhance migration, proliferation, and blood vessel formation, (b) the release of proteases that release growth factors that have been sequestered in the (ECM) to enhance fibroblast proliferation and the angiogenic response and that degrade the ECM, thereby aiding tumor cell invasion of the, (c) contributing to the immune suppressive tumor environment through the release of cytokines such as TGF-β1 and IL-10 and indirectly through interactions with myeloid-derived suppressor cells (MDSC) and regulatory T (Treg) cells. Another known mechanism is tumor-derived TNF-α upregulating PD-L1 expression in the mast cells, representing a mechanism of immune suppression via the direct interaction between MCs and T lymphocytes in a PDL1-dependent manner [12,75].

Scientific studies confirm mast cell pro-tumoral function and the association of Tumor-Associated Mast Cells (TAMCs) with a poor clinical prognosis of various solid tumors. Mostly in the colon [75,76,77,78,79], gastric [80,81,82,83], and pancreatic cancer [84,85,86,87], a growing number of clinical studies have associated high TAMC numbers with tumor progression and worse prognosis in patients. A similar association, though controversial, has been reported for breast [75,88,89,90,91,92], lung [93,94,95], and prostate cancer [96,97]. Furthermore, recent studies showed that human melanoma-associated Mast Cells characterized by an upregulation of the complement component C 3 correlates with poor prognosis [98].

The precise role of MCs in tumor development and progression will be crucial for the development of new targeted therapies in human cancers [99]. The relationship between the MC density of tumors, the progression of angiogenesis, and tumor development may enhance the possible role of MCs in tumor biology. Therefore, the possibility of targeting MC activation [100], inhibiting the release of mediators using c-Kit receptor tyrosine kinase inhibitors (TKI) (imatinib, mastinib, sunitinib [99,101]), or using tryptase inhibitors (gabexate mesylate and nafamostat mesylate, both inhibitors of trypsin-like serine proteases [99,102]) may be valuable therapeutic approaches to control the tumor development [103].

Mastinib is an orally available inhibitor of the protein tyrosine kinase c-kit, which is expressed on the surface of cancer cells. Mastinib also inhibits PDGF and FGF receptors, and fyn and lyn kinases [104,105]. Furthermore, it has been used in veterinary medicine for years, and lately, human clinical trials were initiated to test its clinical efficacy as a single or add-on treatment for human cancers such as mastocytosis, gastrointestinal stromal tumors (NCT00998751), colon cancer (NCT03556956), prostate cancer (NCT03761225), and pancreatic cancer [106].

Imatinib (“Gleevec” or “Glivec”), an orally available tyrosine kinase inhibitor, was called a “magical bullet” when it revolutionized the treatment of chronic myeloid leukemia (CML) in 2001. The first clinical trial of imatinib took place in 1998, and the drug received FDA approval in May 2001. The success of treating patients with CML with imatinib prompted scientists to investigate the therapeutic effect in other types of cancer, and it was found to produce a similar effect in other cancers where tyrosine kinase was overexpressed [101]. Currently, imatinib is the standard of care in CML and GIST as it has dramatically changed the outlook of these diseases. It was the first cancer agent proven effective for metastatic GIST and represented a major development in the treatment of this rare but challenging disease. However, approximately 20% of patients do not respond to imatinib (early or primary resistance), and among those who do respond initially, 50% develop secondary imatinib resistance and disease progression within two years. Patients had no therapeutic option once they became resistant to imatinib before sunitinib was discovered [107].

Sunitinib (“Sutent”) is a small-molecule, multi-targeted (RTK) inhibitor that was approved by the FDA for the treatment of renal cell carcinoma (RCC) and imatinib-resistant (GIST) on 26 January 2006. Sunitinib was the first cancer drug simultaneously approved for two different indications. It offers patients with imatinib-resistant GIST a new treatment option to stop further disease progression and, in some cases, even reverse it. This was shown in a large, Phase III clinical trial in which patients who failed imatinib therapy (due to primary resistance, secondary resistance, or intolerance) were treated in a randomized and blinded fashion with either sunitinib or placebo [107].

Gabexate mesylate is a synthetic serine protease inhibitor that inhibits various kinds of plasma proteins, such as thrombin, plasmin, kallikrein, trypsin, C1 esterase in the complex system, and factor Xa in the coagulation cascade [108,109]. GM inhibits colon cancer growth, invasion, and metastasis by reducing matrix metalloproteinases. The antitumorigenic effect of GM is related in part to the antiangiogenic effect of GM [110].

Nafamostat mesylate is a synthetic serine protease inhibitor; it is short-acting and is also used for the treatment of pancreatitis. Nafamostat also has some potential antiviral and anti-cancer properties [111].

It seems that a combination chemotherapy of tryptase inhibitors or c-Kit receptor inhibitors and classical cytotoxic drugs could potentially exert a synergistic anti-tumor effect. Novel agents killing MCs might be evaluated in adjuvant clinical trials as a new anti-cancer approach.

In addition to the pro-tumoral functions, MCs may also modulate the response of cancer cells to therapy. The in vitro assays demonstrated that MC culture supernatants blocked gemcitabine (GEM)/nabpaclitaxel (NAB)-induced apoptosis in pancreatic cancer cell lines, through the activation of TGF-β1 signaling. Furthermore, these MC-derived supernatants reduced the anti-invasive activity of GEM/NAB. These data showed a functional interplay between MCs and pancreatic cancer cells, which induced resistance to GEM/NAB [75,86]. This observation was supported by the finding that unresponsiveness to GEM/NAB correlated with increased levels of tryptase and TGF-β1 in the blood of pancreatic ductal adenocarcinoma patients. Thus, MCs seem to play a crucial role in tumor resistance to GEM/NAB. Analysis of tumor tissue of inflammatory breast cancer (IBC), an aggressive form of breast cancer characterized by the clinical appearance of inflammation, showed that the MCs degranulating was significantly associated with poor response to neoadjuvant chemotherapy in all disease stages and molecular subtypes of IBC. Moreover, MCs were located within range for direct or paracrine interactions with CD8^+^ T cells, as well as CD163^+^ macrophages and tumor cells. The authors suggested that the interaction of MCs with these immune cells might be exerting an inhibitory effect in IBC, through suppressing CD8^+^ T cells, enhancing immunosuppressive CD163^+^ macrophages, and directly promoting tumor cell growth [75,92]. This study indicated that MCs could represent a possible therapeutic target to enhance the response to chemotherapy.

The summary of the described mast cell-targeted strategies in cancer therapy are presented in Table 3.

## 6. Conclusions

Mast cells are the type of cells that react quickly and rapidly to changes in the environment. As a result of immunological factors or external factors, such as pathogens, proteins, or radiation, they release numerous mediators, changing the homeostasis of the microenvironment surrounding them. MCs degranulation has important physiological roles. These cells participate in innate immunity by recognizing pathogens and then eliminating them. In acquired immunity, mast cells play the role of an immunomodulator of processes, inducing Treg lymphocytes and acting as “non-classical” antigen-presenting cells to T lymphocytes. Excessive activity of mast cells in the responses to external factors such as drugs and food can lead to a dangerous and rapid anaphylactic reaction. The condition is often associated with life-threatening symptoms and in most cases of anaphylaxis does not have a clear, proven cause. MCs are also key cells in the progression of cancer, but research shows that they work in two ways—inhibiting and promoting this process.

Mast cell degranulation can be monitored by a number of tests, such as ELISA, flow cytometry and colorimetric tests. It is possible to track secreted cytokines, chemokines, and other mediators, e.g., histamine, which are crucial in the allergic response.

Inhibiting mast cells degranulation can be achieved by stabilization of the cell membrane, by antihistamines and glucocorticoids. In a more recent approach, still not well studied, are monoclonal antibodies, which target multiple aspects of mast cell functions. Some of them, like omalizumab, are already FDA-approved drugs directed against individual mast cell activators, such as IgE. Many authors emphasized the value of natural-origin substances, like vitamins C, E, and D, and listed them as the first-line drugs for controlling mast cell degranulation. Substances derived from plant extracts and derivatives of fatty acids showed mast cell inhibiting properties too.

Anaphylaxis, as one of the most life-threatening and rapid allergic reactions, requires more predefined procedures. In the first line of defense, it is suggested to use epinephrine, an adrenergic drug acting on α and β receptors. Further management allows for more freedom, and in this area different drugs can be applied. Although many substances, like kinase inhibitors, or even monoclonal antibodies, give very promising results in vitro and in vivo, most of them are still under clinical examination.

Targeting protein kinases is also the main approach inhibiting MCs pro-tumor effects (imatinib, mastinib, sunitinib). As activated mast cells can potentiate the deregulated tissue of the tumor microenvironment and favor tumor growth, it is important to include them in the anti-cancer therapy. It is worth emphasizing that there is also proof that MCs can represent a possible therapeutic target to enhance the response to chemotherapy.

The multiple roles of mast cells in various pathological conditions proves that these cells deserve a special attention as a therapeutical target.

## Figures and Tables

**Table 1:** Compounds and drugs inhibiting mast cell degranulation.

Name of the Drug	Origin or Family of the Drug Molecule	Target	Disease, Animal or Cell Model	References
Olopatadine	Antihistamines	stabilization of cell membrane	various mast cells	[15]
Budesonide	Glucocorticosteroids	stabilization of cell membrane	airway mucosal biopsy specimen from human	[19]
Omalizumab	Monoclonal antibodies	IgE	FDA-approved drug	[20]
Ligelizumab		IgE	clinical trials	
Dupilumab		interleukin signaling	FDA-approved drug	
MTPS9579A		tryptase	clinical trials	
CDX-0159		c-kit/CD117 receptor	clinical trials	
Vit. C	Vitamins	inhibiting histidine decarboxylase activity (the enzyme responsible for the production of histamine from histidine)	blood, urine, and tissue (lung, gastric mucosa, and spleen) of guinea pigs; blood serum of human patients with allergic and non-allergic diseases	[21]
Vit. D		binding to the gene transcription apparatus (regulating gene expression of selected mediators like TNF-α)	Animal models: Male BALB/c mice, Sprague Dawley rats; Cell lines: human mast cell line HMC1, rat mast cell line RBL-2H3, mouse mast cell lines p815, and mouse BMMCs	[23]
Fisetin, kaempferol, myricetin, quercetin, and rutin	Flavonoids	precise target unknown (inhibited histamine release and intracellular calcium ions)	rat mast cells RBL-2H3	[27]
Salvinorin A	Diterpenoid	precise target unknown (reduced release of β-hex, histamine, IL-4 and TNF-α)	rat mast cells RBL-2H3	[29]
Coptisine	Alkaloids	precise target unknown (decreased levels of β-hex, histamine, IL-4 and TNF-α)	rat mast cells RBL-2H3	[30]
Curcumin, monomethylcurcumin and bisdemethoxycurcumin	Curcuminoids	precise target unknown (reduced release of β-hex and inhibition of IL-4 and TNF-α production)	rat mast cells RBL-2H3; BALB/c mice and mouse BMMCs	[26,32]
Docosahexaenoyl ethanolamide	Derivatives of fatty acids	IgE-mediated degranulation (decreased release of β-hex and calcium ions influx)	rat mast cells RBL-2H3	[33]
Anandamide		FcεRI-dependent degranulation (reduced intracellular calcium ions)	mouse BMMCs	[34]
Palmitoylethanolamide		precise target unknown (reducing serotonin, histamine, β-hex and NGF)	rat mast cells RBL-2H3	[35,37,38]

**Table 2:** Drugs for anaphylactic shock treatment.

Name of the Drug	Origin or Family of the Drug Molecule	Target	Disease, Animal or Cell Model	References
Epinephrine	Adrenergic drug	Adrenergic receptors: α1 receptor α2 receptor β1 receptor β2 receptor	Emergency treatment for acute allergic reactions (anaphylaxis) caused by peanuts or other foods, medications, insect bites and stings, and other allergens, as well as exercise-induced or idiopathic anaphylaxis.	[47,48,67]
Albuterol		β2 adrenergic receptor	Prevention and treatment of difficulty breathing, wheezing, shortness of breath, coughing, and chest tightness caused by asthma and chronic obstructive pulmonary disease.	[49]
Dupilumab	Monoclonal antibody	IL-4Rα	Atopic dermatitis.	[50,51]
Sirtuin 6	NAD^+^-dependent deacetylase	Suppressor of PTPRC (Protein tyrosine phosphatase, receptor-type C) transcription	Murine bone marrow-derived mast cells (BMMCs), Human cord blood-derived mast cells, Myeloid Sirt6 KO mice (Sirt6*flox/flox*; LysM-Cre) Mast cell-deficient KitW-sh/W-sh mice	[52]
Ibrutinib	Bruton’s tyrosine kinase inhibitors	Bruton’s tyrosine kinase	Mantle cell lymphoma, chronic lymphotic leukemia and Waldenstroms macroglobuline-mia. Clinically tested for food-induced anaphylaxis.	NCT03149315 [53]
Acalabrutinib			As monotherapy or in combination with obinutuzumab, it is indicated for the treatment of adult patients with previously untreated chronic lymphocytic leukemia. Clinically tested for food-induced anaphylaxis.	NCT05038904 [53]
Tirabrutinib			Treatment of recurrent or refractory primary central nervous system lymphoma and under review for the treatment of Waldenström’s macroglobulinemia and lymphoplasmacytic lymphoma. For anaphylaxis treatment tested on primary human skin-derived mast cells (SDMCs).	[53]
Anemoside B4	PLC/IP3 and JAK/STAT3 pathways inhibitor	The exact target requires further research.	Laboratory of allergic disease 2 (LAD2) cell line and in vivo mice model.	[55]
Fucoxanthin	Natural compound	The exact target requires further research.	BALB/c mice	[56,57]
Benzoylpaeoniflorin				[58]
Ginsenosides	AKT-NrF2 and p38MAPK-Nrf pathways inhibitor	The exact target requires further research.	Cell lines: HMC-1 (human mast cell line) RBL-2H3 (rat basophilic leukemia cell line) RAW 264.7 cells (murine macrophages) RBL 2H3 cells (rat basophils) Animals: Male Hartley guinea pigs Male ICR mice SD rats	[60,61,62]
Alpha-linolenic acid	Lyn kinase inhibitor	Lyn kinase	Laboratory of allergic disease 2 (LAD2) cell line C57BL/6 wild-type (WT) mice	[65]
Salicylaldehyde			Passive anaphylaxis mouse model using C57BL/6J and BALB/c	[66]

**Table 3:** Mast cell-targeted strategies in cancer therapy.

Name of the Drug	Origin or Family of the Drug Molecule	Target	Disease, Animal or Cell Model	References
Mastinib	c-Kit tyrosine kinase inhibitor	Protein tyrosine kinase c-kit expressed on the surface of cancer cells PDGF and FGF receptors Fyn and lyn kinases	Mastocitosis Gastrointestinal stromal tumors Colon cancer Prostate cancer Pancreatic cancer	[81,101,104,105,106] NCT00998751 NCT03556956 NCT03761225
Imatinib			Chronic myeloid leukemia Gastrointestinal stromal tumor	[81,101,107]
Sunitinib			Renal cell carcinoma Gastrointestinal stromal tumor	[81,101,107]
Gabexate mesylate	Serine protease inhibitor	Thrombin Plasmin Kallikrein Trypsin C1 esterase Coagulation factor Xa	Colon cancer	[81,102,108,109,110]
Nafomast mesylate		Prothrombin Coagulation factor X Coagulation factor XII Trypsin Kallikrein Intercellular adhesion molecules (ICAM)	Pancreatitis Antiviral and anti-cancer properties	[81,102,111]

## Data Availability

Data sharing not applicable.
